# Efficacy and safety of artificial pneumothorax with position adjustment for CT‐guided percutaneous transthoracic microwave ablation of small subpleural lung tumors

**DOI:** 10.1111/1759-7714.13137

**Published:** 2019-07-09

**Authors:** Haipeng Jia, Jie Tian, Bo Liu, Hong Meng, Fengmin Pan, Chunhai Li

**Affiliations:** ^1^ Department of Radiology Qilu Hospital of Shandong University Jinan China; ^2^ Department of Respiration Huantai County Hospital of Traditional Chinese Medicine Zibo China

**Keywords:** Artificial pneumothorax, lung tumors, microwave ablation, pain relief

## Abstract

**Background:**

To evaluate the efficacy and safety of artificial pneumothorax with position adjustment for computed tomograpy (CT)‐guided percutaneous transthoracic microwave ablation (MWA) of small subpleural lung tumors.

**Methods:**

Fifty‐six patients with small subpleural lung tumors (< 3.0 cm) entered the study and underwent CT‐guided MWA with (group I: 24 patients with 24 tumors) or without (group II: 32 patients with 34 tumors) the support of artificial pneumothorax. Follow‐up contrast‐enhanced CT scans were reviewed. Pain VAS (visual analog scale) scores at, during, and after ablation were compared between the two groups. Technical success, technique efficacy, local tumor control and complications were compared.

**Results:**

Creation of the artificial pneumothorax was achieved for 24/24 (100%) in group I and no complication related to the procedure was observed. Technical success of MWA was achieved for all 58 tumors. Primary efficacy of MWA was achieved in 23 of 24 tumors (95.8%) treated in group I, and 32 of 34 tumors (94.1%) treated in group II (*P* = 0.771). The 12‐month local tumor control was achieved in 87.5% (21/24) in group I compared with 88.2% (30/34) in group II (*P* = 0.833). Pain VAS scores in group I were significantly decreased after the pneumothorax induction at, during, and after ablation compared with group II (*P* < 0.05). There was no significant difference in MWA‐related complications (*P* > 0.05).

**Conclusion:**

Artificial pneumothorax with position adjustment for CT‐guided MWA is effective and may be safely applied to small subpleural lung tumors. Artificial pneumothorax is a reliable therapy for pain relief.

## Introduction

Lung cancer is currently the worldwide leading cause of cancer‐related mortality, accounting for one‐quarter of all cancer deaths.^1^ Surgical resection remains the reference standard for the treatment of localized non‐small cell lung cancer. However, only 20% of all diagnosed lung cancers are surgically resectable.^2^ Thermal ablative therapy with radiofrequency ablation (RFA) and microwave ablation (MWA) has been successfully used to locally control and palliate thoracic neoplasms, including early‐stage primary lung cancer and pulmonary metastases.^3^, ^4^, ^5^, ^6^, ^7^

RFA, the most commonly used technique, has an 80%–90% reported rate of complete ablation, with the best results obtained for lung tumors <3.0 cm in diameter.^3^ However, the success of RFA in the lung is limited by the low conductivity of the aerated lung, and the heat‐sink effect of adjacent blood vessels and airways.^8^ An alternative ablation technique in which MWA is used provides all of the benefits of RFA and substantial advantages. In contrast to RFA, MWA has a higher conduction and a lower heat‐sink effect in the lung tissues, thus permitting larger ablation zones.^9^ Studies have shown that MWA is evolving into a suitable alternative and adjuvant method to open thoracic surgery of primary and metastatic lung cancer, which is commonly associated with high morbidity and mortality.^7^, ^10^

However, one of the major issues associated with MWA is damage to neighboring critical structures. When MWA is performed on tumors located under the pleura or near the chest wall, intercostal nerves are potentially at risk. Injury to nerves can lead to neuropathic pain. The parietal pleura and chest wall are sensitive to pain because abundant sensory nerve branches originate from the intercostal nerves, contrary to the case in the visceral pleura and lung parenchyma.^11^ Pain experienced by patients during and after MWA of subpleural lung tumors is an important concern. Severe intraprocedural pain may oblige operators to alter the ablation planning (e.g., decrease microwave power and ablation time) to reduce pain. Such altered planning may affect the ablation zone and, ultimately, local efficacy. Several separation techniques have been described to separate the ablation zone from adjacent structures before ablation, including placement of fluid or free air.^12^ However, the small sample size, single‐center‐based case reports, and the lack of evaluation of local control are important limitations of most reports.^13^, ^14^, ^15^, ^16^

The purpose of our study was to evaluate the efficacy and safety of artificial pneumothorax with position adjustment for computed tomography (CT)‐guided percutaneous transthoracic MWA of small subpleural lung tumors, including pain relief, safety and local tumor control.

## Methods

### Study population

We obtained approval from the institutional review board for performing artificial pneumothorax allowing CT‐guided percutaneous MWA of lung tumors. Written informed consent was obtained from all participating patients before performing this procedure.

Between June 2016 and January 2018, 407 patients who were confirmed to have pulmonary tumors following enhanced CT/PET‐CT and lung biopsy, underwent CT‐guided percutaneous MWA. Tumors in all patients were deemed medically inoperable, or the patients had refused surgery. Indication for performing artificial pneumothorax for CT‐guided MWA of subpleural lung tumors was the distance from tumor to pleura <3.0 cm.^14^, ^16^ For curative ablation, the size of the ablation zone is always larger than the targeted tumour. When the target tumor‐pleura distance is less than 3.0 cm, the ablation zone may cover the pleura and is damage to neighboring pleura, which may be associated with a high possibility of severe pain. The exclusion criteria for this study were as follows: (i) known contraindications of thermal ablation^17^; (ii) history of ipsilateral thoracic surgery or radiation therapy and (iii) lung tumor size >3.0 cm in diameter.

A total of 56 patients entered the study. Twenty‐four patients with 24 subpleural lung tumors were treated by MWA under local anesthesia with the support of artificial pneumothorax (group I). Thirty‐two patients with 34 subpleural lung tumors were treated by MWA without artificial pneumothorax (group II). Characteristics of patient group I were as follows: 15 men, nine women; mean age 65 years ±17 (standard deviation); range, 26–81 years. Characteristics of patient group II were as follows: 22 men, 10 women; mean age 60 years ±15; range, 28–82 years. The patient demographic and baseline characteristics of the two groups is summarized in Table 1.

**Table 1:** Patient demographic and baseline characteristics

Characteristics	Group I (*N* = 24)	Group II (*N* = 32)	*P*‐value
Age (years)*	65 ± 17	60 ± 15	0.304†
Gender (male)	15 (62.5)	22 (68.8)	0.625‡
BMI (kg/m^2^)*	26.4 ± 4.1	27.1 ± 5.4	0.161†
Smoker	13 (54.2)	19 (59.4)	0.697‡
Tumor size (cm)*	2.0 ± 0.8	2.2 ± 0.9	0.073†
Tumor location			0.970‡
Upper and middle lobes	14 (58.3)	20 (58.8)	
Lower lobe	10 (41.7)	14 (41.2)	
Pleural to lesion depth (cm) *	1.2 ± 0.7	1.3 ± 0.9	0.630†
Lesion type			0.899‡
Solid	18 (75.0)	25 (73.5)	
Ground‐glass	6 (25.0)	9 (26.5)	
Tumor characteristics			0.896‡
Primary lung cancer	11 (45.8)	15(44.1)	
Metastatic tumors	13 (57.1)	19 (55.9)	
From colorectal cancer	6 (25.0)	8 (23.5)	
From liver cancer	3 (12.5)	5 (14.7)	
From breast cancer	2 (8.3)	2 (5.9)	
From kidney cancer	2 (8.3)	4 (11.8)	
Procedure characteristics			
Power (W)*	51.4 ± 5.2	49.8 ± 6.2	0.530†
Time (min)*	4.7 ± 1.1	4.8 ± 1.6	0.722†

* Data are mean ± standard deviation.

† *P*‐value was calculated with the independent sample *t*‐test.

‡ *P*‐value was calculated with the Pearson χ^2^test.

Unless otherwise indicated, data are numbers of patients or tumors, and data in parentheses are percentages.

### Procedures

Artificial pneumothoraxes were created on all patients before MWA. MWA in all patients was performed with CT guidance (SOMATOM Force, Siemens Healthcare, Germany), with 5 mm collimation, 110 reference kVp and 50 reference mAs using automated tube current modulation (CAREDose4D). Patients received conscious sedation with continuous blood pressure, electrocardiography and oxygen saturation monitoring during the entire procedure. All procedures were performed by a single radiologist (C.L.) with 12 years experience in CT‐guided interventional procedures and five years experience in MWA.

#### Pneumothorax induction

**Figure 1:**
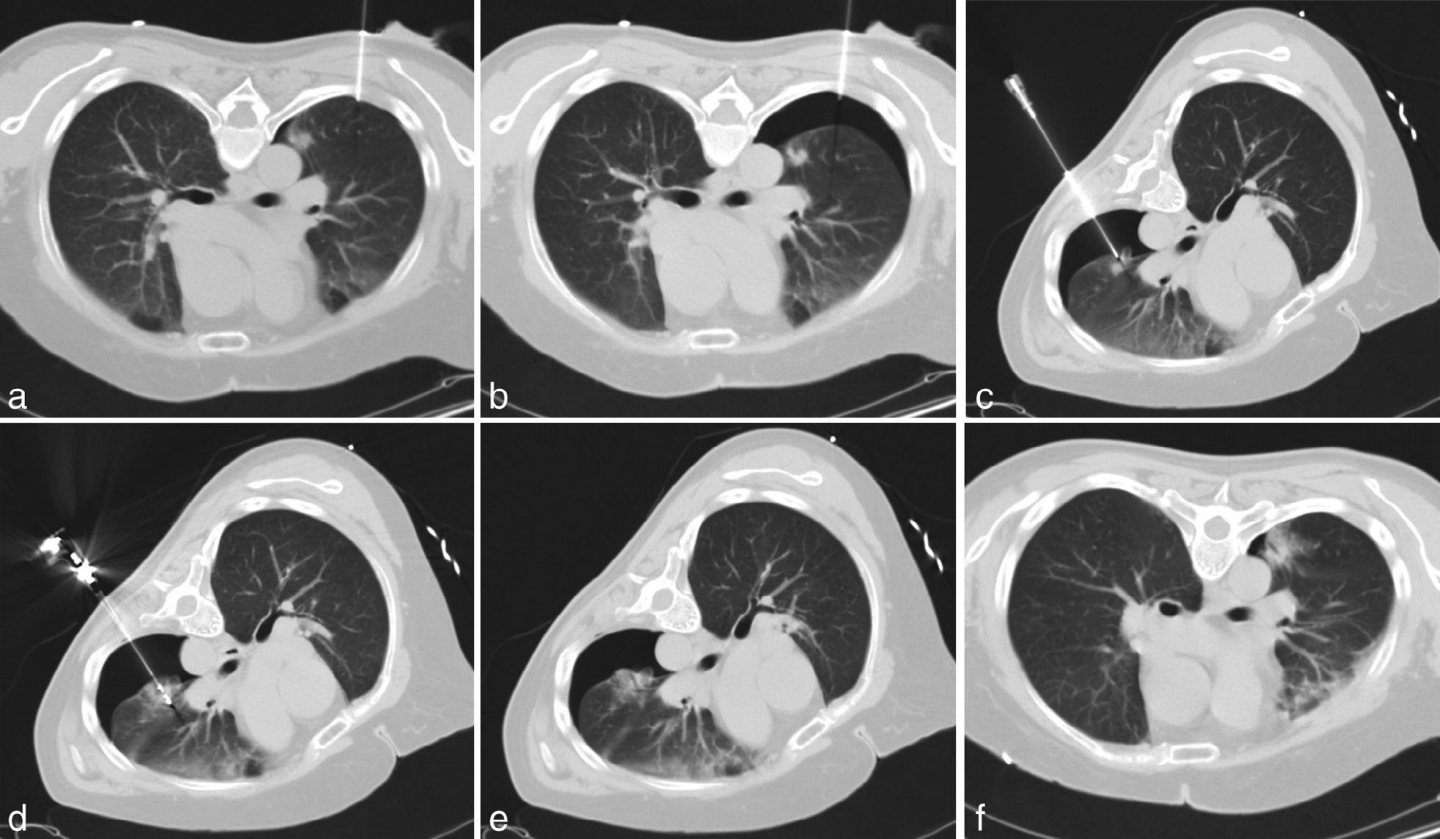
This shows a 62‐year‐old woman with biopsy‐proven 1.7 cm adenocarcinoma of the lower lobe of the left lung treated with MWA. (**a**) An 18‐gauge puncture needle was inserted into the chest wall. (**b**) Air was injected into the pleural space to make the artificial pneumothorax. (**c**) Following successful artificial pneumothorax with position adjustment the biopsy needle tip was located precisely in the nodule. (**d**) Image during MWA showed single microwave antenna positioned with its tip at the distal portion of tumor (50 W, 5 minutes). (**e**) Images obtained immediately after ablation showed perilesional ground‐glass opacification induced by MWA therapy. (**f**) Most of the air was aspirated from the pleural space after MWA, leaving a small residual pneumothorax.

Patients were placed in a supine or prone position, depending on the location of the tumor, as indicated by CT scan. The techniques used to create an artificial pneumothorax are shown in Figure 1. Based on the scan images, puncture sites were chosen in the CT scanning plane containing the tumor or placed adjacent to the peripheral tumor. The perpendicular distances from puncture sites to pleura parietal layer were measured. Local anesthesia at the entry site was given by injections of 1% lidocaine hydrochloride. An 18‐gauge puncture needle (BioPrince, Argon Medical, USA) was inserted into the chest wall to gain percutaneous access to the pleural space. A syringe without piston was injected into 5 mL normal saline connected to the puncture needle. The needle was progressed slowly along the direction perpendicular to the chest wall. When the saline liquid level fell due to the negative intrapleural pressure, it indicated that the needle tip had entered the pleural cavity. Five millilitre of air was injected and the location of the needle tip was verified with CT. If the needle had not reached the pleural space on the CT image, it was pushed forward again until the tip reached it. Once the needle had reached the pleural space, a 0.035‐inch guidewire (RF‐GA35153M; Terumo Co., Tokyo) was then threaded through the needle into the pleural cavity to exchange the needle for a 6‐F pigtail catheter (Angiotech SKATER), which was connected to a three‐way stopcock and a 50 mL syringe. The syringe was withdrawn to ensure that no blood return occurred. Fifty millilitre air obtained through the stopcock was then injected into the pleural space through the catheter. A CT scan was performed to make sure that the artificial pneumothorax had been produced. A total of 150–‐200 mL air was injected once to expand the pleural cavity. The position of each patient was then adjusted to make the lesion as high as possible in the thoracic cavity, so that the target lesion could be separated from the pleura with minimal air injection (the distance from tumor to chest wall >1.0 cm).

#### Microwave ablation

Treatment plan, puncture site, and the best entry route of the microwave antenna were designed according to CT images after the artificially induced pneumothorax. Lidocaine was administered at the puncture site to carry out local anesthesia of the pleura. With CT monitoring, an ablation antenna was inserted through the chest wall and then through the empty pleural cavity and finally reached the targeted tumor. MWA was performed using a microwave applicator (ECO‐100A1, Microwave Electronic Institute, Nanjing, China) at the 2.45 GHz frequency designated for medical use. The applicator included a 16‐gauge straight microwave antenna (10, 15 or 20 cm length). A peristaltic pump perfused the outer shaft of the antenna with normal saline at a rate of 60 mL/minute to prevent thermal injury along the proximal antenna shaft. The manufacturer's recommendations (4–7 minutes for 3.7 cm active tip at 60 W, 4–7 minutes for 3.2 cm active tip at 55 W or 50 W and 4–6 minutes for 2.7 cm active tip at 45 W, respectively) were adhered to in all cases. Ablation time and power were determined according to the tumor size, geometry and location and recorded for all procedures. A single session at one single point was usually adequate to achieve complete ablation of small lesions (< 3.0 cm).^7^, ^17^ After completion of the MWA session, the ablation antenna was removed. In addition, percutaneous needle lung biopsy, if necessary, was administered before MWA. The detailed lung biopsy procedure is described in our latest study.^18^

#### Pneumothorax removal

After MWA, intrapleural air was extracted through the deposited catheter into the syringe and expelled through the stopcock. This maneuver was repeated until no more air could be aspirated. Another chest CT examination covering the entire chest was carried out to confirm if there was any residual pneumothorax. In addition, a 24 hour post‐procedural CT was obtained to eliminate the delayed pneumothorax. The pneumothorax progressed and was then considered nonartificial.

### Complications assessment

All patients underwent 24‐hour post‐procedural CT to eliminate the delayed development of complications. In accordance with the Common Terminology Criteria for Adverse Events (CTCAE) v4.0 of the National Cancer Institute, complications were categorized as major and minor complications, and side effects.^19^ Major complications led to substantial morbidity and disability, and required interventional drainage procedure or blood transfusion to be performed. The main side effects were pain, post‐ablation syndrome, and asymptomatic minor bleeding or fluid accumulation on CT. All other complications were considered to be minor.

Visual analog scale (VAS) scores were assessed and recorded for pain measurements during the MWA procedures and at 6, 12, and 24 hours after the procedures. Intraprocedural severe pain was treated using local anesthesia together with epidural anesthesia.

### Follow‐up imaging evaluation

Follow‐up contrast‐enhanced CT was performed at 1‐, 3‐, and 6‐month intervals after the initial ablation session. After contrast enhancement, both pattern and magnitude of CT attenuation were used to evaluate the extent of thermocoagulation. Complete lack of enhancement in the ablation zone indicated adequate ablation of the tumor. A thin symmetric rim of peripheral enhancement of less than 5 mm wide observed up to six months after ablation was considered a sign of benign peritumoral inflammatory response. Irregular focal soft‐tissue enhancement (> 15 HU) was considered to be a sign of residual or recurrent disease.^5^, ^20^ In patients in whom local tumor progression was found on initial postablation CT scans or subsequent follow‐up images (evaluated by H.J. and B.L.), reablation was performed. Progressive disease in the ablated region was retreated between one and 12 months after initial ablation.

### Data collection

Patients were classified into two groups, on the basis of the ablation procedure with or without artificial pneumothorax (group I and group II). Patient demographics (sex, age, and body mass index), characteristics of target lesions (tumor size, location, type and distance from pleura to target lesion), and procedure‐related information (procedure time and power) were recorded (Table 1).

Evaluation included the following variables: technical success rates (tumors were treated according to protocol and were covered completely by the ablation zone^19^); primary efficacy rates (the percentage of target tumors successfully eradicated at three month interval after initial ablation), and local tumor control, which was defined by the absence of both local tumor residue and progression. Side effects and complications, such as pain, pleural effusion, and pneumothorax were recorded on a per‐treatment basis. In group I, the amount of air and the thickness of artificial pneumothorax were also recorded.

## Statistical analysis

Analyses were performed using MedCalc 12.7.4 (MedCalc Software, Ostend, Belgium). Continuous variables were expressed as the mean ± standard deviation (mean ± SD). Categorical variables were expressed as frequency and the percentage of patients. The differences between two groups were assessed using the Student *t*‐test or Wilcoxon rank‐sum test for continuous data and Chi‐square test or the Fisher exact test for categorical data. All statistical tests were two‐tailed; a *P*‐value <0.05 was considered statistically significant.

## Results

### Pneumothorax induction

Induction of artificial pneumothorax was successfully achieved in all 24 patients (group I). The procedure was well tolerated by all patients as evidenced by maintenance of oxygen saturation greater than 90%, and no abnormal fluctuations in blood pressure. The mean time required to establish the artificial pneumothorax with the positional adjustment was 17.5 minutes (range, 9–34 minutes). Injection of 100–550 mL (mean, 356 mL) of air was required. The artificial pneumothorax was 1.0–2.8 cm thick (mean thickness, 1.7 cm) at the level of the tumors on CT images during MWA. The tumors pulled by the artificial pneumothorax stayed >1.0 cm away from the chest wall.

### Effectiveness

A single microwave antenna was used in all patients in 58 ablation sessions. Technical success of MWA (tumors that were treated according to protocol) was achieved for all 58 tumors, with each application lasting 3.5–5 minutes (mean time, 4.1 ± 1.0 minutes).

In group I, primary efficacy rate of MWA was achieved for a total of 23 of 24 tumors (95.8%). In the group II protocol, primary efficacy rate was achieved in 32 of 34 tumors (94.1%). No difference in primary technique efficacy was found between the two groups (*P* = 0.771).

The 12‐month local tumor control rate was achieved with the group I protocol in 21 of 24 tumors (87.5%), and with the group II protocol in 30 of 34 tumors (88.2%). Local tumor control rate showed no significant difference between group I and group II (*P* = 0.833). The time to tumor progression or residual detection after MWA for two groups is shown in Table 2. The median time to local tumor progression treated with the group I protocol was six months and 4.5 months for tumors treated with the group II protocol, with no statistically significant difference noted.

**Table 2:** Time to local tumor recurrence or residual tumor detection

Months after MWA	Group I (*n* = 24)	Group II (*n* = 34)	*P*‐value
3	1 (4.2)	2 (5.9)	0.771
6	2 (8.3)	1 (2.9)	0.361
12	0 (0)	1 (2.9)	0.398
Total	3 (12.5)	4 (11.7)	0.833

Data are numbers of tumors, and data in parentheses are percentages. MWA, microwave ablation; Group I, MWA with artificial pneumothoraxes; Group II, MWA without artificial pneumothoraxes.

### Side‐effects and complications

No intraprocedural deaths occurred in any of the groups. Patients in the group II protocol suffered from significant pain during and after ablation procedures. In group I, the mean VAS scores were 1.43 ± 0.44, 1.8 ± 0.39, 2.1 ± 0.51, and 0.75 ± 0.32 at during‐procedure, 6, 12, and 24 hours, respectively. In group II, the mean VAS scores were 4.61 ± 1.90, 2.77 ± 1.53, 3.43 ± 1.88, and 1.83 ± 0.56 at during‐procedure, 6, 12, and 24 hours, respectively. Pain VAS scores were significantly different at during‐procedure, 6, 12 and 24 hours between the two groups (*P* < 0.05) (Table 3). In group II, six patients (18.75%) were treated by using local anesthesia together with epidural anesthesia due to unacceptable moderate to severe pain at the beginning of MWA. Eight patients (25.0%) in group II experienced significant postprocedure pain (VAS scores: 4–6) localized to the site of ablation. Oral administration of 50 mg of meperidine and intramuscular injection of 25 mg hydroxyzine were required for pain control whilst the patients were in recovery.

**Table 3:** Pain VAS scores evaluation in all patients

VAS score	Group I (*N* = 24)	Group II (*N* = 32)	*P*‐value
During‐procedure	1.43 ± 0.44	4.61 ± 1.90	< 0.001
6 h	1.8 ± 0.39	2.77 ± 1.53	0.023
12 h	2.1 ± 0.51	3.43 ± 1.88	0.001
24 h	0.75 ± 0.32	1.83 ± 0.56	0.001

Data are mean ± standard deviation. VAS, visual analog scale.

Pneumothorax occurred in six of 24 patients (25%) in the group I protocol, and 11 of 32 patients (34.4%) in the group II protocol (Table 4). Pneumothorax classified as a minor complication was treated conservatively with no interference (in four of 24 patients [16.7%] treated with group I, and seven of 32 patients [21.9%] treated with group II). Pneumothorax classified as a major complication was treated with a chest tube placement in two of the 24 patients (8.3%) in group I and four of the 32 patients (12.5%) in group II. No statistically significant difference was noted between two groups in terms of pneumothorax.

**Table 4:** Frequency of MWA‐related complications

Complications	Group I (*N* = 24)	Group II (*N* = 32)	*P*‐value
Pain*	0 (0)	8 (25.0)	0.008
Pneumothorax	6 (25.0)	11 (34.4)	0.450
Minor†	4 (16.7)	7 (21.9)	0.627
Major‡	2 (8.3)	4 (12.5)	0.617
Pleural effusion	11 (45.8)	17 (53.1)	0.589
Pulmonary hemorrhage	7 (29.2)	12 (37.5)	0.514

* Pain necessitated drug therapy after MWA.

† Treated with no interference.

‡ Treated with chest tube.

Data are numbers of patients, and data in parentheses are percentages.

Pleural effusion was the most common side effect that occurred in 45.8% (11 of 24) of patients in group I compared with respective incidence of 53.1% (17 of 32) in group II (*P* = 0.589). Pulmonary hemorrhage occurred in 29.2% (seven of 24) of patients and hemoptysis occurred in 4.2% (one of 24) in the group I compared with respective incidence of 37.5% (12 of 32) and 6.3% (two of 32) in the group II (*P* = 0.514 and *P* = 0.631).

## Discussion

This study sought to investigate the feasibility of artificial pneumothorax with position adjustment for CT‐guided percutaneous transthoracic MWA of small subpleural lung tumors. It introduced a simple procedure to build an artificial pneumothorax using regular instruments applied to MWA in patients. The results indicated that artificial pneumothorax with position adjustment is an effective and safe method for CT‐guided MWA of small subpleural lung tumors. Furthermore, an artificial pneumothorax can obviously effect pain relief during and after MWA of subpleural lung tumors.

MWA is a minimally invasive treatment option for patients with primary and metastatic pulmonary malignancies in nonsurgical candidates.^5^, ^6^, ^7^ However, the literature on the percutaneous ablation of lung tumors is still incomplete, and some technical issues need to be addressed and optimized. One of the main issues with MWA of subpleural lung tumors is pain management during and after the procedure. In particular, patients with targeted lesions involving the pleura and/or chest wall (pleural to lesion depth < 1.0 cm) may experience unendurable pain during and after ablation under local anesthesia, usually requiring general anesthesia to proceed with the ablation procedure.^21^, ^22^ MWA‐induced post‐procedural pain may lead to difficult and prolonged recovery in patients.^14^ Therefore, MWA may become less cost‐effective and less attractive as an alternative therapeutic method to surgery.

In this study, we adopted the artificial pneumothorax as a technique for pain relief during and following the ablation procedure. Pain VAS scores in group I were significantly decreased after pneumothorax induction during ablation and postablation compared with group II. The results suggest that an artificially induced pneumothorax can significantly mitigate the pain for patients undergoing MWA of small subpleural lung tumors. Similar findings have also been found in previous case reports.^13^, ^14^, ^15^, ^16^ In our study, artificial pneumothorax separated the tumor and surrounding pulmonary tissue from the pleura and the chest wall, which contributed to effectively protect the intercostal nerve branches and thus relieve chest pain. We used an 18‐gauge puncture needle to obtain percutaneous access to the pleural space, following the theory that normal saline liquid level decrease is induced by negative pressure in the pleural cavity. This method was safe and reliable for judgment of the timing of puncturing the needle into the pleural cavity. Thus, no unpredicted complication related to the procedure was observed in our study.

Large amounts of air in the pleural space can impair respiratory function, leading to discomfort for patients. Therefore, it is important to control and minimize the amount of air that is injected when generating an artificial pneumothorax. We demonstrated that adjustment of the patient's position enabled the operator to inject a smaller volume of air into the patient's thorax. In our study, the mean volume of injected air was 356 mL (range, 100–550 mL), which was notably less than the 750 mL air injection reported by Lee *et al*.^14^ All patients in group I had no hemodynamic or oxygenation change during the insufflation procedure.

In this study, artificial pneumothorax for CT‐guided MWA of small subpleural lung tumors (group I) showed a primary efficacy rate of 95.8% (23 of 24) and local tumor control of 87.5% (21/24). These results were comparable to the overall technique efficacy of MWA of lung tumors without artificial pneumothorax (group II). Tumors of less than 3.0 cm were completely ablated in 78%–96% of cases according to previous reports.^5^, ^6^, ^7^, ^23^ Technique efficacy for MWA of small tumors (≤ 3.0 cm) in this study compared favorably with those reports. Furthermore, we observed that the time to local tumor progression rates were comparable in two groups. In general, our findings indicated that artificial pneumothorax for CT‐guided MWA had good local control for the treatment of small subpleural lung tumors.

With regard to complications of MWA of lung tumors, no significant difference was found between the two groups. In our study, pneumothorax occurred in 25% (six of 24) of ablations, with a chest tube required in 8.3% (two of 24) of ablations in group I, similar to group II for MWA. Pneumothorax was less common than previously reported in the literature. Belfiore *et al*. reported a pneumothorax rate of 32% in patients treated with MWA, with a chest tube required in 14% of cases.^24^ The most common side effects noted in the present study were asymptomatic pleural effusions.

In conclusion, this study demonstrates that artificial pneumothorax with position adjustment for CT‐guided MWA is effective and may be safely applied to small subpleural lung tumors. Artificial pneumothorax is a reliable therapy for pain relief and as a result of this study, creation of an artificial pneumothorax for MWA of subpleural lung tumors is now routinely used in our institution.

## Disclosure

No authors report any conflict of interest.
